# Change in fatty liver status and 5-year risk of incident metabolic syndrome: a retrospective cohort study

**DOI:** 10.1186/s40885-015-0032-7

**Published:** 2015-11-17

**Authors:** Eun Na Han, Eun Sun Cheong, Jeong In Lee, Min Chul Kim, Christopher D. Byrne, Ki-Chul Sung

**Affiliations:** Department of Medicine, Kangbuk Samsung Hospital, School of Medicine, Sungkyunkwan University, Seoul, Republic of Korea; Division of Cardiology, Department of Medicine, Kangbuk Samsung Hospital, School of Medicine, Sungkyunkwan University, Seoul, Republic of Korea; Nutrition and Metabolism, Faculty of Medicine, Southampton National Institute for Health Research, Biomedical Research Centre, University Hospital Southampton, University of Southampton, Southampton, UK

**Keywords:** Fatty liver disease, Fatty liver, Change in fatty liver, Metabolic syndrome, Insulin resistance

## Abstract

**Introduction:**

Fatty liver is associated with metabolic syndrome (MetS) but it may also occur without MetS. Whether resolution of fatty liver in the general population affects risk of MetS is unknown. Our aim was to determine whether a change in fatty liver status (either the development of new fatty liver or the resolution of existing fatty liver) would modify the risk of de novo MetS.

**Methods:**

Two thousand eighty-nine people without hypertension, diabetes, and MetS were examined at baseline and at 5-year follow-up using a retrospective cohort study design. Fatty liver status was assessed at baseline and at follow-up by ultrasonography. Adjusted hazard ratios (aHR) and 95 % confidence intervals (CIs) for de novo MetS at follow-up were calculated controlling for the potential confounders, compared to the reference group (people who never had fatty liver at baseline and follow-up).

**Results:**

During follow-up, fatty liver developed in 251 people and fatty liver resolved in 112 people. After the adjustment for multiple confounders, persisting fatty liver and incident fatty liver development were associated with de novo MetS, with aHR of 2.60 (95 % CIs [1.61,4.20]) and 3.31 (95 % CIs [1.99,5.51]), respectively. Risk of new MetS in resolved fatty liver group was attenuated with insignificant aHR of 1.29 accompanying 95 % CIs of 0.60 and 2.80.

**Discussion:**

Development or maintenance of fatty liver is positively associated with occurrence of new MetS. Resolution of fatty liver status has similar risk of de novo MetS with those who never had fatty liver. Therefore, cautious management is needed with those with fatty liver.

## Introduction

Non-alcoholic fatty liver disease (NAFLD) is defined as a disorder with excess fat in the liver due to non-alcoholic causes [1]. NAFLD is recognized as the most common cause of liver disease worldwide, with a prevalence of 15–35.8 % in Western populations [2–6], 14–20 % in Japanese [7], and is more frequent in people with increased amounts of body fat, occurring in up to 85 % in overweight individuals and 98 % non-diabetic obese individuals [8].

The metabolic syndrome (MetS) is a cluster of cardiometabolic disorders which is known be a risk factor for development of atherosclerotic cardiovascular disease and stroke [9]. It occurs in association with central obesity and insulin resistance. The major components of MetS include insulin resistance, central obesity, dyslipidemia, and hypertension [10]. Increased plasma glucose and triglyceride concentrations are key components of metabolic syndrome which are overproduced in NAFLD. The liver is therefore a key determinant of these metabolic abnormalities [1]. NAFLD and MetS are observed often in the same individual, and insulin resistance is assumed to play a key role that links them together. It is reported that nearly 90 % of NAFLD patients have more than one component of metabolic syndrome [11], but it is also known that NAFLD can occur in people who do not have insulin resistance and features of MetS [12].

The severity of fatty liver is associated with increased cardiovascular risk factors including Mets [13]; however, it is uncertain whether change in fatty liver status, either development of new fatty liver or resolution of existing fatty liver, affects the development of metabolic syndrome or not. We have previously investigated relationships between change in fatty liver status and incident hypertension/diabetes [14, 15]. However, in these studies, we were not able to adjust waist circumference which was not available on the whole cohort. Since it is not known whether change in fatty liver status is associated with development of MetS, in this study, we have analyzed the relationship between change in fatty liver status and development of incident MetS at 5-year follow-up.

## Methods

### Study population

The study population consisted of individuals who had a comprehensive health medical examination at baseline (in 2003) and were re-examined 5 years later (in 2008) at Kangbuk Samsung Hospital, College of Medicine, Sungkyunkwan University, South Korea. In South Korea, employees are required to participate in annual or biennial health examinations by the industrial safety and health law. A total of 2174 participants who had waist circumference data and without hypertension, diabetes, and metabolic syndrome at baseline were included initially. Individuals with data missing at baseline for the following variables were excluded: alcohol consumption (*n* = 42), smoking (*n* = 41), and exercise (*n* = 18). Some patients were excluded for more than one reason. Therefore, 2089 participants were eligible for this analysis.

The examinations were performed without any selection of high-risk individuals for differential testing. The institutional review board at Kangbuk Samsung Hospital has approved the secondary analysis of anonymized data from the cohort for this study. Informed consent was not required because personal identifying information was not used.

### Measurement

The health examination included full medical histories, blood samples, physical examinations, anthropometry, and abdominal ultrasonography. Body mass index (BMI) was calculated as weight in kilograms divided by height in meters squared. Questionnaires were given to examine to ascertain information regarding alcohol consumption (g/day), smoking (never, ex, current), and frequency of exercise (none, less than once a week, at least once a week). Blood samples were collected after at least 10 h of fasting and analyzed in the same core clinical laboratory. The core clinical laboratory has been accredited and participates annually in inspections and surveys by the Korean Association of Quality Assurance for Clinical Laboratories. Triglyceride, high-density lipoprotein cholesterol (HDL-C) and fasting plasma glucose, AST, and ALT were measured using Bayer Reagent packs on an automated chemistry analyzer (Advia 1650 Autoanaylizer; Bayer diagnostics, Leverkusen, Germany). Intra- and inter-assay coefficients of variation for all biochemical measurements were <5 %. Insulin concentration was measured with an immunoradiometric assay (Biosource, Nivelle, Belgium) with an intra and inter-assay coefficient of variation of 2.1–4.5 and 4.7–12.2 %, respectively. Insulin resistance was assessed with the homeostatic model assessment-insulin resistance (HOMA-IR) according to the following equation: Fasting blood insulin (mU/ml) × Fasting blood glucose (mmol/l)/22.5.

Abdominal ultrasonography (Logic Q700 MR; GE, Milwaukee, WI, USA) was conducted by clinical radiologists using a 3.5-MHz probe for all subjects at baseline and after 5 years. The following images were undertaken: (1) sagittal view of the right lobe of the liver and right kidney, (2) transverse view of the left lateral segment of the liver and spleen, and (3) transverse view of the liver for altered echo texture. Fatty infiltration of the liver (fatty liver) was identified if there was an increase in echogenicity of the liver compared with the echogenicity of the renal cortex where the diaphragm and intrahepatic vessels appeared normal [16].

The metabolic syndrome definition was used from the National Cholesterol Education Program (NCEP/ATP III) [10]. Current ATP III criteria defined the metabolic syndrome as the presence of any three of the following five traits: waist circumference in men ≥90 cm and in women ≥80 cm in Asian patient, serum triglycerides ≥1.7 mmol/L (150 mg/dL) or drug treatment for elevated triglycerides, serum HDL-C <1 mmol/L (40 mg/Dl) in men and <1.3 mmol/L (50 mg/dL) in women or drug treatment for low HDL-C, blood pressure ≥130/85 mmHg or drug treatment for elevated blood pressure, and fasting plasma glucose (FPG) ≥5.6 mmol/L (100 mg/dL) or drug treatment for elevated blood glucose.

### Statistical analysis

Continuous variables were indicated as mean ± SD for normally distributed variables or median and interquartile range if variables were not normally distributed. ANOVA and independent *t* test were performed to compare continuous variables, and non-normally distributed variables were compared via Mann-Whitney U and Kruskal-Wallis tests. Logistic regression was used to determine the hazard ratio (HR) for metabolic syndrome at follow-up regarding never fatty liver group as the reference: (a) in patients with resolution of fatty liver over 5 years, i.e., fatty liver that had been present at baseline, but was not present at follow-up examination; (b) in patients with the development of new fatty liver at follow-up examinations; and (c) in patients with fatty liver that was present at both baseline and at follow-up. Analyses were undertaken with the following adjustments. Model 1 was adjusted for age and sex; model 2 for the same risk factors as model 1 plus alcohol consumption, smoking status, and exercise; and model 3 for the same risk factors as model 2 plus glucose, waist, systolic blood pressure, triglyceride, and HDL-C.

All data were analyzed using PASW statistics 18.0 (IBM, Armonk, New York, USA). The statistical significance was defined as *p* value <0.05 (two-tailed).

## Results

Two thousand eighty-nine participants were enrolled for this study, and amongst these subjects, 159 developed incident metabolic syndrome at 5-year follow-up.

Table 1 shows the baseline characteristics of groups stratified by the change of fatty liver status: (1) no fatty liver at baseline nor at follow-up (reference group), (2) fatty liver at baseline but no fatty liver at follow-up (resolved fatty liver group), (3) no fatty liver at baseline but fatty liver at follow-up (incident fatty liver group), and (4) fatty liver at both baseline and at follow-up (persisting fatty liver group). Glucose, triglyceride, HDL-C, BMI, blood pressure, fasting insulin level, hepatic enzymes, and HOMA-IR were positively associated with the change of fatty liver status (*p* value <0.05, *p* for trend <0.05).

**Table 1:** Baseline characteristics of four groups stratified according to the change in fatty liver status

Variables	No fatty liver—no fatty liver (reference group) (*n* = 1344)	Fatty liver—no fatty liver (resolved fatty liver) (*n* = 112)	No fatty liver—fatty liver (incident fatty liver) (*n* = 251)	Fatty liver—fatty liver (persisting fatty liver) (*n* = 382)	*P* value	*P* for trend
Males (*n*, %)	822 (61.2 %)	101 (90.2 %)	215 (85.7 %)	361 (94.5 %)	<0.001	
Age	40.9 ± 6.5	41.9 ± 6.7	41.1 ± 5.6	41.5 ± 5.7	0.210	
Glucose (mmol/L)	4.91 ± 0.44	5.05 ± 0.45	5.00 ± 0.44	5.09 ± 0.45	<0.001	0.002
Triglyceride (mmol/L)	1.05 [0.79, 1.42]	1.63 [1.28, 2.09]	1.62 [1.13, 2.06]	1.79 [1.37, 2.42]	<0.001	<0.001
HDL-C (mmol/L)	1.54 ± 0.31	1.38 ± 0.25	1.42 ± 0.28	1.36 ± 0.23	<0.001	<0.001
Waist circumference (cm)	76.8 ± 8.3	86.2 ± 5.6	82.6 ± 5.9	87.6 ± 5.3	<0.001	<0.001
BMI (kg/m^2^)	22.6 ± 2.6	25.2 ± 2.2	24.1 ± 2.2	25.7 ± 2.0	<0.001	<0.001
SBP (mmHg)	109.6 ± 10.1	114.2 ± 8.0	112.4 ± 9.2	114.2 ± 7.8	<0.001	<0.001
DBP (mmHg)	70.5 ± 8.0	73.5 ± 6.4	72.0 ± 7.4	74.0 ± 6.3	<0.001	<0.001
Alcohol	10.45 ± 14.20	11.13 ± 12.44	13.67 ± 16.29	12.27 ± 14.90	0.005	0.367
Smoking status					<0.001	<0.001
Never smoker	765 (56.9 %)	37 (33.0 %)	96 (38.2 %)	113 (29.6 %)		
Current smoker	579 (43.1 %)	75 (67.0 %)	155 (61.8 %)	269 (70.4 %)		
Exercise ≥1/week	555 (41.3 %)	41 (36.6 %)	90 (35.9 %)	125 (32.7 %)	0.014	<0.001
Incident diabetes development	1 (0.1 %)	2 (1.8 %)	3 (1.2 %)	6 (1.6 %)	0.001	<0.001

Data are (*n*, %) or mean ± SD or median [IQR]. *P* value was calculated by the ANOVA and independent *t* test for continuous variables, and Mann-Whitney U and Kruskal-Wallis tests for non-normally distributed variables

*BMI* body mass index, *SBP* systolic blood pressure, *DBP* diastolic blood pressure

In each of the four groups, we assessed the numbers and percentages of individuals in each of the respective group who developed incident MetS at follow-up. These data showed 41 (3.1 % of the group at baseline) for the reference group, 10 (8.9 % of the group at baseline) for the resolved group, 36 (14.3 % of the group at baseline) for the incident group, and 72 (18.8 % of the group at baseline) for the persisting group (Table 2).

**Table 2:** Prevalence of metabolic syndrome components in the four groups at baseline and at follow-up

Metabolic syndrome components	Fatty liver status (*n* = 1344)		Fatty liver status (*n* = 112)		Fatty liver status (*n* = 251)		Fatty liver status (*n* = 382)	
	Baseline	Follow-up	Baseline	Follow-up	Baseline	Follow-up	Baseline	Follow-up
	No fatty liver	No fatty liver	Fatty liver	No fatty liver	No fatty liver	Fatty liver	Fatty liver	Fatty liver
	*n* (%)	*n* (%)	*n* (%)	*n* (%)	*n* (%)	*n* (%)	*n* (%)	*n* (%)
Waist circumference, (men ≥90 cm/women ≥80 cm)	72 (5.4)	110 (8.2)	27 (24.1)	16 (14.3)	28 (11.2)	46 (18.3)	110 (28.8)	108 (28.3)
Triglyceride (TG ≥1.7 mmol/L or drug treatment)	215 (16.0)	213 (15.8)	50 (44.6)	40 (35.7)	112 (44.6)	123 (49.0)	205 (53.7)	202 (52.9)
HDL-C (HDL-C <1 mmol/L (men)/<1.3 mmol/L (women) or drug treatment)	90 (6.7)	100 (7.4)	6 (5.4)	12 (10.7)	19 (7.6)	45 (17.9)	20 (5.2)	52 (13.6)
Blood pressure (BP >130/85 mmHg or drug treatment)	69 (5.1)	172 (12.8)	8 (7.1)	28 (25.0)	16 (6.4)	53 (21.1)	24 (6.3)	99 (25.9)
Glucose (glucose ≥5.6 mmol/L or drug treatment)	106 (7.9)	232 (17.3)	16 (14.3)	37 (33.0)	20 (8.0)	73 (29.1)	59 (15.4)	131 (34.3)
Metabolic syndrome (presence of any three of the following five traits)	0 (0 %)	41 (3.1 %)	0 (0 %)	10 (8.9 %)	0 (0 %)	36 (14.3 %)	0 (0 %)	72 (18.8 %)

Data are (*n*, %)

We examined the prevalence of MetS components at baseline and at follow-up in each of the four groups (Table 2). In the resolved fatty liver group, the proportion of people with increased triglyceride and waist circumference decreased at follow-up compared with that at baseline measurements. In contrast, proportion of people with components of MetS increased in the incident fatty liver group. The prevalence of MetS was highest in persisting fatty liver group (18.8 %), and it was higher in incident fatty liver group (14.3 %) than the resolved fatty liver group (8.9 %).

Table 3 shows hazard ratios for incident MetS at follow-up, according to the change in fatty liver status. Persisting fatty liver and incident fatty liver development were associated with incident MetS, even after the adjustment for multiple confounders with aHR of 2.60 (95 % CIs [1.61, 4.20]) and 3.31 (95 % CIs [1.99, 5.51]), respectively. Risk of incident MetS was attenuated with insignificant aHR of 1.29 accompanying 95 % CIs as 0.60 and 2.80 in “resolved fatty liver group.”

**Table 3:** Hazard ratios (HRs) for incident metabolic syndrome according to the fatty liver status at baseline and at follow-up

	HR [95 % CI]^a^			
	No fatty liver—no fatty liver (reference group) (*n* = 1344)	Fatty liver—no fatty liver(resolved fatty liver) (*n* = 112)	No fatty liver—fatty liver (incident fatty liver) (*n* = 251)	Fatty liver—fatty liver (persisting fatty liver) (*n* = 382)
Model 1	1	3.05 [1.47–6.36]	5.34 [3.29–8.65]	7.34 [4.77–11.28]
Model 2	1	3.10 [1.48–6.47]	5.31 [3.27–8.63]	7.42 [4.80–11.47]
Model 3	1	1.29 [0.60–2.80]	3.31 [1.99–5.51]	2.60 [1.61–4.20]

Model 1: adjusted for age and sex; model 2: adjusted for model 1 plus alcohol, smoking status, and exercise; model 3: adjusted for model 2 plus glucose, waist circumference, systolic blood pressure, triglyceride, and HDL-C

^a^Logistic regression was used to determine hazard ratio (HR) for developing metabolic syndrome at follow-up

Adjustment of changes in every metabolic component is illustrated in Table 4. Risk of incident MetS became insignificant with those adjustments in all groups.

**Table 4:** Associations between incident MetS and fatty liver status derived from a multivariable logistic regression model containing all variables

	HR (95 % CI)	*P* value
Reference group	1	0.81
Resolved fatty liver	0.90 (0.33,2.40)	0.83
Incident fatty liver	1.26 (0.68.2.35)	0.46
Persisting fatty liver	1.22 (0.69.2.14)	0.50
Age	0.99 (0.95.1.02)	0.48
Sex	6.21 (2.58.14.92)	<0.001
Alcohol	1.00 (0.99.1.01)	0.84
Smoking	0.83 (0.48.1.43)	0.50
Exercise	1.19 (0.74.1.90)	0.47
Glucose	1.12 (1.09.1.16)	<0.001
Triglyceride	1.01 (1.01.1.01)	<0.001
HDL-C	0.89 (0.86.0.92)	<0.001
Waist circumference	1.16 (1.11.1.21)	<0.001
Systolic blood pressure	1.10 (1.06.1.13)	<0.001
Change in waist circumference	1.23 (1.16.1.31)	<0.001
Change in SBP	1.09 (1.07.1.11)	<0.001
Change in glucose	1.10 (1.07.1.13)	<0.001
Change in TG	1.01 (1.01.1.01)	<0.001
Change in HDL-C	0.90 (0.86.0.93)	<0.001

## Discussion

Our novel results show that change in fatty liver status over 5 years is associated with risks of developing MetS at 5-year follow-up. The development of incident fatty liver during the 5-year follow-up period was associated with an increased hazards ratio for incident MetS, and there was marked attenuation of the risk of MetS with resolution of fatty liver, which showed similar risk with the reference group indicating that those without MetS would be more likely to develop MetS if she/he has new or persistent NAFLD.

As shown in Table 2, the components of metabolic syndrome have been improved in “resolved fatty liver group” but worsened in the “incident fatty liver group.” Therefore, NAFLD can be considered as alternative way to describe MetS even though this is not included as a component of MetS. Definite pathogenic mechanism of MetS is not clear, and several definitions of MetS exist [17]. As the current definitions of MetS do not reflect the entire MetS itself, diagnosis of MetS with NAFLD would predict metabolic status or risk of certain individuals more precisely. This is the novel data of our study conducted with individuals without baseline MetS. The major significance of this study is that those without MetS would mostly proceed to MetS if she/he has persistent NAFLD. Thus, cautious management of this kind of subjects is needed.

The mechanism which fatty liver contributes to MetS is not properly understood but regulation of hepatic lipid metabolism may be dissociated from the regulation of glucose metabolism. For example, overexpression of diacylglycerol acyltransferase 2 (DGAT2) which catalyzes the final step in triacylglycerol (TG) biosynthesis in the liver increases hepatic steatosis, manifested as increased amounts of hepatic TG, diacylglycerol, ceramides, and unsaturated long-chain fatty acyl-CoAs. Mice overexpressing DGAT2 did not have abnormalities of glucose tolerance or insulin levels [18], supporting the notion that hepatic steatosis may not necessarily be caused by insulin resistance.

Why does change in fatty liver status modify risk of developing MetS? One theory addressing this question involves the role of hepatokines and inflammatory cytokines that are secreted by the liver which may modulate metabolic risk and insulin resistance [19, 20]. The liver secretes many hepatokines. For example, plasminogen-activator inhibitor-1 is an inflammatory protein that is secreted by the liver and may affect risk of type 2 diabetes [21]. Ardigo et al. found that plasma concentration of plasminogen-activator inhibitor-1 was only elevated in individuals with both evidence of insulin resistance and ultrasound-diagnosed fatty liver and not insulin resistance alone [22]. And inflammatory biomarkers such as C reactive protein, tumor necrosis factor α, and interleukin-6 may directly affect risk of some of the MetS components by adversely affecting hepatic gluconeogenesis, glycogen synthesis, and insulin signaling [23–26]. These results might support our results.

Resolution and development of fatty liver disease varies between individuals and is linked to many different factors which could affect risk of MetS. For example, variations in diet, physical activity, fluxes of fatty acids, hepatic oxidative stress, cytokine production, reductions in very low-density lipoprotein secretion, and alterations in the intestinal microbiome are all associated with changes in NAFLD [26, 27]. Additionally, increases in free fatty acids (FFA), interleukin (IL)-6, tumor necrosis factor (TNF)-alpha, together with other pro-inflammatory cytokines that occur with adipose tissue inflammation and changes in adipose tissue function are also associated with insulin resistance [28].

Our study has some limitations. Ultrasound has limited sensitivity to detect low levels of fatty liver and to detect fatty liver in very obese subjects. However, in this Asian cohort, there were very few subjects with a BMI greater than 30 kg/m^2^ (males 14 and females 3). Furthermore, in this cohort, it was not possible to assess agreement between radiologists in reporting hepatic steatosis diagnosed by ultrasound, as the ultrasound examination was undertaken by 18 different sonographers within the routine clinical service. But inter-observer reliability and intra-observer reliability for fatty liver diagnosis were considered substantial (kappa statistic of 0.74) and excellent (kappa statistic of 0.94), respectively, at another study which was conducted in the same clinic center in 2011 [29]. Finally, we did not consider the severity of NAFLD in each liver group when classifying the participants, as the numbers in each group was large enough to obtain meaningful results.

## Conclusion

Development or maintenance of fatty liver is positively associated with occurrence of new MetS. Resolution of fatty liver status has similar risk of de novo MetS with those who never had fatty liver. Therefore, NAFLD is important as an alternative way to present MetS despite not included as a component of MetS, and cautious management is needed with those with fatty liver.

## Abbreviations

ALT: alanine aminotransferase
AST: aspartate aminotransaminase
BMI: body mass index
DBP: diastolic blood pressure
GGT: γ-glutamyltransferase
HDL-C: high-density lipoprotein cholesterol
LDL-C: low-density lipoprotein cholesterol
MetS: metabolic syndrome
NAFLD: non-alcoholic fatty liver disease
SBP: systolic blood pressure
TG: triglyceride
